# A Closed-Loop Autologous Erythrocyte-Mediated Delivery Platform for Diabetic Nephropathy Therapy

**DOI:** 10.3390/nano12203556

**Published:** 2022-10-11

**Authors:** Lingzi Feng, Xinzhong Huang, Jia Li, Chao Chen, Yidan Ma, Haiying Gu, Yong Hu, Donglin Xia

**Affiliations:** 1 School of Public Health, Nantong University, Nantong 226019, China; 2 Department of Nephrology, Affiliated Hospital of Nantong University, Nantong 226001, China; 3 MOE Key Laboratory of High Performance Polymer Materials & Technology, College of Engineering and Applied Sciences, Nanjing University, Nanjing 210033, China

**Keywords:** diabetic nephropathy, hemodialysis, closed loop, insulin, erythrocyte

## Abstract

Failure to control blood glucose level (BGL) may aggravate oxidative stress and contribute to the development of diabetic nephropathy (DN). Using erythrocytes (ERs) as the carriers, a smart self-regulatory insulin (INS) release system was constructed to release INS according to changes in BGLs to improve patients’ compliance and health. To overcome the limited sources of ERs and decrease the risk of transmitting infections, we developed an in vitro, closed-loop autologous ER-mediated delivery (CAER) platform, based on a commercial hemodialysis instrument modified with a glucose-responsive ER-based INS delivery system (GOx-INS@ER). After the blood was drained via a jugular vein cannula, some of the blood was pumped into the CAER platform. The INS was packed inside the autologous ERs in the INS reactor, and then their surface was modified with glucose oxidase (GOx), which acts as a glucose-activated switch. In vivo, the CAER platform showed that the BGL responsively controlled INS release in order to control hyperglycemia and maintain the BGL in the normal range for up to 3 days; plus, there was good glycemic control without the added burden of hemodialysis in DN rabbits. These results demonstrate that this closed-loop extracorporeal hemodialysis platform provides a practical approach for improving diabetes management in DN patients.

## 1. Introduction

Diabetic nephropathy (DN), also known as diabetic kidney disease, is a typical kidney disease (abnormal renal function and/or the presence of persistent albuminuria) occurring as a result of diabetes mellitus (DM) [1,2,3]. Along with the prevalence of diabetes, the number of patients suffering DN has been growing year by year [4,5,6,7]. At worse, the efficacy of current treatments for DN is unsatisfactory, and more than 65 % of end-stage renal disease patients require hemodialysis [8]. Despite the control of the uremic symptoms, glycemic control is essential for DN therapy [9,10,11]. Disappointingly, most patients with a long duration of diabetes and poor glycemic control tend to accelerate the development of progressive DN [12]. Therefore, a consistently effective therapeutic strategy with good glycemic control and eliminated uremic symptoms is urgently required for this disease.

The aim of insulin (INS) therapy is to mimic the physiological secretion of INS; continuous subcutaneous injections of INS are required for glycemic control in DN because of its short half-life. Although effective in short periods, frequent injection cannot mimic the normal physiological INS secretion pattern upon variation in the BGL [13,14,15,16]. Even with the combinations of short- and long-term INS releasing systems, complications, such as intermittent hypoglycemic periods of excessive INS and hyperglycemic periods with a lack of INS, may occur if the doses and timings of INS are imprecise [17,18,19,20]. Ideally, INS therapy should provide an appropriate quantity of INS to restore the BGL [21,22,23].

Due to the ability to modulate the pharmacokinetic or biological activity of carried payloads, erythrocytes (ERs), ghost ERs, or ER-mimetic nanoparticles have been extensively explored as drug deliverers [24,25,26]. Li et al. developed an ER-hitchhiking drug delivery system, which extended the systemic circulation of the attached drugs and specifically dropped off the payloads via inflammation-responsive means [27]. Our previous study also demonstrated that INS-loaded glucose oxidase (GOx) modified erythrocytes (GOx-INS@ERs) could extend the systemic circulation of INS and self-regulate the release of INS upon changes in the BGL [28]. In this system, large amounts of gluconic acid and hydrogen peroxide were produced as the BGL increased, which promoted the release of INS from GOx-INS@ERs within several minutes. Animal studies revealed that GOx-INS@ERs had a good glycemic control effect for up to 9 days in diabetic rats. Unfortunately, the origin of the blood remains a major obstacle for success in in vivo studies and clinical applications [29]. So, due to the inconvenient intravenous injections (must be operated by a trained professional) and limited ER sources (blood products are regulated in many countries), this INS delivery system, similar to other drug-loaded ER systems, cannot be applied in the clinic in its current form (as in vitro isolated and modified ERs are needed) [30,31,32]. It is recognized that this design needs to be improved to realize the in vivo application.

Hemodialysis is the most common treatment, routinely performed three times a week at a hemodialysis center [33]. For commercial hemodialysis, blood is removed through a dialysis catheter located in the right atrium or superior vena cava, externally filtered, and then re-supplied to the blood through the same catheter [34,35]. The patient’s bloodstream is connected to the hemodialysis machine with a closed-loop dialysis vascular access for hemodialysis therapy. This is a safe purification technique for curing renal failure.

**Scheme 1:**
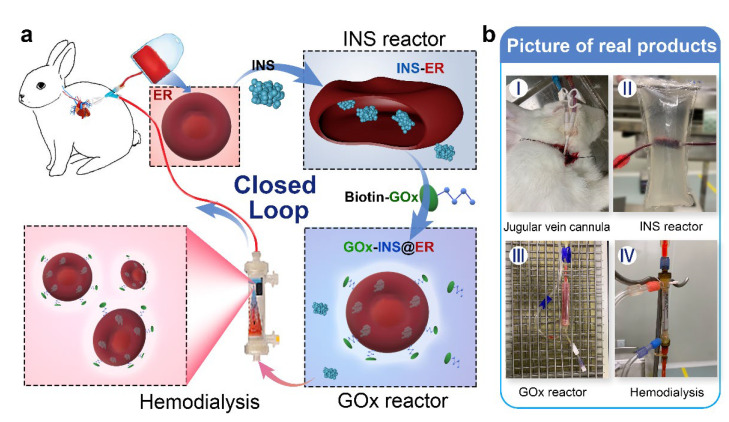
Schematic representation of the in vitro, closed-loop autologous ER-mediated delivery (CAER) platform. (**a**) The schematic representation shows the CAER platform, accomplished by the construction of a glucose-responsive, self-regulated INS release system (GOx-INS@ERs). First, blood was drained via a jugular vein cannula and eventually put back into the animal’s vein. INS was loaded into ERs in the INS reactor by the hypotonic dialysis method. Second, GOx was modified on the surface of the INS@ERs in the GOx reactor. Finally, GOx-INS@ER solution was dialyzed to remove residual drugs before being returned to the venous blood. (**b**) Pictures of actual products, including the jugular vein cannula, INS reactor, GOx reactor, and hemodialysis equipment.

Inspired by the fact that closed-loop extracorporeal blood circulation would overcome the limitation of blood sources (using autologous ERs as the INS carrier) and decrease the risk of transmitting infections, we constructed an in vitro, INS-loaded closed-loop autologous ER-mediated delivery (CAER) platform (Scheme 1 and Figure S1). It enables the blood purification with a commercial hemodialysis instrument, while accomplishing the construction of the glucose-responsive, self-regulated INS release system (GOx-INS@ERs) for DN treatment. In this system, the blood was drained via a jugular vein cannula after heparin administration. The hemodialysis was performed, and a proportion of the ERs was chosen as the INS-carrier. Next, the GOx was modified on the surface of the ERs for the intelligent control of INS release based on the BGL. Finally, the GOx-INS@ER solution was dialyzed to remove the free INS and residual GOx and transported back into the body of the DN rabbit. We anticipate that this system can combine the hemodialysis treatment and glycemic control simultaneously, without increasing the treatment burden, thus improving the life quality of DN patients.

## 2. Material and Methods

### 2.1. Chemicals and Animals

INS from bovine pancreas (acidity ≥ 27 U/mg), biotin N-hydroxysuccinimide ester, GOx, and alloxan were obtained from Bomei Biotechnology Co., Ltd. (Hefei, China). Dialysis bags were supplied by Green Bird Science & Technology, Inc. (Shanghai, China) (molecular weight cut-off: 10 kDa and 5 kDa). *FITC*-labeled INS (*FITC*-*INS*) was provided by Zhongke Chenyu Biotechnology (Beijing, China). Heparin sodium and ulatan were purchased from Aladdin (Shanghai, China). Rabbit anti-CD55 antibody (bs-1552R), rabbit anti-CD59 antibody (bs-1638R), and mouse anti-rabbit IgM/FITC antibody (bs-0369M-FITC) were from BIOSS (Beijing, China). Other reagents were from Sigma-Aldrich.

Female rabbits (3–4 kg) were provided by the Experimental Animal Center of Nantong University. All animals were housed in individual cages and maintained under standardized conditions. All animal experiments were performed under the guidance of relevant laws and regulations examined by the Division of Comparative Medicine of Nantong University (S20210416-004). 

### 2.2. Preparation of Dialysate

A certain amount of INS powder was dissolved in HCl solution at pH 2 and the pH was adjusted to 7.2–7.4 with Na_2_HPO_4_ to obtain the fresh INS solution. The hypotonic buffer (72 mOsm/kg), hypertonic solution (550 mOsm/kg), and isotonic solution (300 mOsm/kg) used in the INS reactor were prepared in advance according to the previous study [36]. The exact recipes for these solutions are summarized in Supplementary Table S1. The commercially available dialysate was provided by Shandong WEGO pharmaceutical Co., Ltd., Shandong, China. 

### 2.3. Establishment of the DM/DN Rabbit Model

After overnight fasting, female rabbits were intraperitoneally injected with alloxan (100 mg/kg, body weight) to induce diabetes. Three days later, the rabbits (plasma glucose levels > 20 mmol/L) were used as DM models in the following experiments [37]. 

Each DN rabbit model was built on the DM model and fed with an additional high cholesterol diet for 2 weeks [37,38]. The urine (at 24 h) and venous blood of DM rabbits were collected and the 24 h urine protein level, serum blood urea nitrogen (BUN), and serum creatinine (CREA) concentrations were measured. The 24 h urine protein > 30 mg, BUN > 17 mmol/L, and CREA > 171 μmol/L were regarded as the successful establishment of the DN model [38].

### 2.4. Establishment of the CAER Platform

The CAER platform was built based on the commercial hemodialysis equipment (DBB-27, WEGO, Shangdong, China) with some modifications. This platform included three major parts: the INS reactor, the GOx reactor, and Nano-A hemodialyzer with hollow fibers. A schematic of the CAER platform is shown in Figure S1.

#### 2.4.1. The INS Reactor Design and Fabrication

The INS reactor was designed by our group and constructed in Shandong Weihai WEGO Blood Purification Products Co., Ltd. As shown in Scheme 1B II and Figure S2, the dialysis bag (molecular cut-off, 10 kDa) was mounted on the inside of a sterile plastic bag. The blood could be drained into the INS reactor through catheter A and drained out through catheter B. The hypotonic buffer and hypertonic or isotonic solution could be perfused in through catheter C and out through catheter D. The inlet, catheter A, remained on the rubber plug to add INS to the inner dialysis bag (Figure S2 inset).

#### 2.4.2. Design and Manufacturing of the GOx Reactor and Hemodialyzer

The GOx reactor was designed as a sterile tube that was tied in place with clips to control fluid injection. For the convenience to inject drugs, the GOx reactor was designed with an injection inlet (Scheme 1B III).

The Nano-A hemodialyzer was manufactured and generously supplied by the manufacturer (WEGO, Shangdong, China).

#### 2.4.3. The CAER Platform Set Up

Before connecting the hemodialysis equipment and the CAER platform, the rabbits were firstly anesthetized with 20% sodium ulatan (3–4 mL/kg) and treated with 400 U/kg heparin for systemic heparinization. Blood was drained via a jugular vein cannula into the hemodialysis equipment and eventually back into the animal’s vein through an indwelling trocar. During the entire dialysis period, the CAER platform worked together with the hemodialysis machine.

##### Synthesis of INS@ERs in the CAER Platform

Blood was drained via a jugular vein cannula into the hemodialysis equipment and about 2 mL of blood was drained into the dialysis bag of the INS reactor dialysis bag as the INS solution was injected into the dialysis bag. Then, a syringe was used to push INS into the INS reactor through the rubber plug. The temperature of the hypotonic buffer was adjusted to 0, 4, 10, 27, or 37 °C, and circulated in the INS reactor by a peristaltic pump at a 200 mL/min flow rate. After 4 h, the hypotonic buffer (37 °C) was replaced by hypertonic solution and circulated in the INS reactor for another 0.5 h at 200 mL/min. Finally, the isotonic solution (room temperature) was drained into the INS reactor for another 15 min to restore the ERs to their normal size. After the above procedure, the INS was encapsulated in ERs, obtaining INS@ERs.

Different conditions (including INS content, dialysis time, and dialysis temperature) were tested to optimize the encapsulation experiment: (1) INS content: 12.5, 25, 50, 75, or 100 U with a dialysis time 4 h and hypotonic buffer temperature set at 4 °C. (2) Dialysis time: 0.5, 1, 2, or 4 h; hypotonic buffer temperature: 4 °C; administrated INS content: 75 U. (3) Dialysis temperature: 0, 4, 27, or 37 °C; dialysis time: 4 h; and administrated INS content: 75 U.

##### Preparation of GOx-INS@ERs

The obtained INS@ERs in the INS reactor were drained into the GOx reactor by PBS injection through the rubber plug of the INS reactor. A total of 200 μL Na_2_B_4_O_7_·10H_2_O (0.1 M) and 6 μL biotin-NHS (0.1 M) was added to the GOx reactor and incubated for 20 min. Then, different amounts of biotin-GOx were added into the GOx reactor and shaken for 30 min with a Laboratory Shaker-HY-4 (Langyue, China) at a speed of 90–100 cpm. INS@ERs and GOx in different proportions were tested to optimize the encapsulation experiment. Finally, the mixture was pushed into the hollow fibers of the hemodialyzer to remove unloaded INS and unmodified GOx. The obtained GOx-INS@ER suspension was eventually transported back into the animal vein. The activity of immobilized GOx on INS@ERs was measured using a conventional GOx assay (Bomei, Hefei, China). The loading efficiency of INS in ER was calculated as follows: drug loading efficiency (%) = weight of loaded INS/ weight of feeding INS × 100%. 

### 2.5. Characterization

#### 2.5.1. Scanning Electron Microscopy (SEM)

The ERs in the INS reactor during the hypotonic, hypertonic, and isotonic processes were collected, and their morphologies were characterized by SEM (JSM-6700F microscope). SEM also characterized the morphologies of GOx-INS@ERs in different concentrations of glucose solution. The collected samples were treated with 2.5% (*v*/*v*) glutaraldehyde in PBS (0.1 M, pH 7.4) for 3 h at 4 °C. After being washed with distilled water at room temperature, they were sprayed with gold, and SEM images were obtained.

#### 2.5.2. Fluorescence Characterization

To further demonstrate that the INS was encapsulated in the ERs and the GOx was on the surface of GOx-INS@ERs, the rhodamine-B-labeled GOx and FITC-INS were used to prepare GOx-INS@ERs. Laser confocal pictures of GOx-INS@ERs were captured using a Leica TCS SP8 STED 3X microscope (Leica, Mannheim, Germany).

#### 2.5.3. Flow Cytometric Analysis

The difference in CD55 and CD59 expression between natural ERs and GOx-INS@ERs was analyzed by flow cytometry. In brief, 2 × 10^5^ of cells were incubated with rabbit anti-CD55 antibody or rabbit anti-CD59 antibody at 37 °C for 30 min followed by mouse anti-rabbit IgM/FITC antibody at 37 °C for 30 min. After staining, the cells were analyzed by flow cytometry (FACSCalibur, BD Biosciences).

#### 2.5.4. INS Concentration Measurement

The INS concentrations in the INS@ERs, GOx-INS@ERs, and in vivo experiments were tested as Shao et al. described [14]. In brief, 100 μL of carrier ERs was taken and added to the HCl solution (0.1 mol/L). The INS could be fully released from the ER and dissolved. An INS ELISA kit was used to detect the INS concentration (GSB-E05071m, Cusabio, Wuhan, China).

### 2.6. Hematological Parameters Analysis

The hematological parameters of INS@ERs in the INS reactor and GOx-INS@ERs in the GOx reactor were determined via a HEMAVET 950FS hematological system analyzer. Hematocrit value (HCT), hemoglobin concentration (HGB), mean corpuscular volume (MCV), and mean corpuscular hemoglobin (MCH) were chosen as the blood biochemistry index and analyzed. Blood levels of BUN and CREA and serum levels of calcium (Ca^2+^) ions and potassium (K^+^) concentrations were detected via colorimetry (Bomei Bioengineering Institute, Hefei, China).

### 2.7. In Vitro Glucose-Responsive INS Release from GOx-INS@ERs

The in vitro INS release behavior of GOx-INS@ERs (after hemodialysis) was carried out in a simulated-use test mimicking a DN patient. A total of 1 mL of GOx-INS@ER suspension was drawn from the CAER platform and soaked into 10 mL of saline at 37 °C with 15 mmol/L of D-glucose (simulating a hyperglycemic environment). 

After centrifugation, the GOx-INS@ERs were resuspended again in 10 mL 5 mmol/L of D-glucose (corresponding to normal glucose level) for another 30 min. GOx-INS@ERs in the hyperglycemic environment were defined as “on” and in normoglycemia solution were defined as “off”. The INS@ERs or the GOx-INS@ERs, collected after hemodialysis, were immersed in 5 mmol/L or 15 mmol/L of D-glucose solution for 5 min alternatively and the INS concentrations in the supernatant were tested.

The GOx-INS@ER membrane blocking degrees were tested according to the report by Zamudio [39]. In brief, after the GOx-INS@ERs had been in the hyperglycemic and normoglycemia environments, the concentrations of NADHs were measured. The membrane blocking degree was calculated as previously recommended [39].

### 2.8. In Vivo Testing of the CAER Platform

According to the principles of maximization and the results of the INS loading content, 75 U of administrated INS content, 4 °C dialysis temperature, and 2 mg/mL GOx were adopted as the optimized procedure. A total of 10 DM and 10 DN rabbits were chosen and randomly divided into 4 groups (5 rabbits per group). The treatment groups were: (1) DM: diabetic rabbits, set as control, (2) DN + HD: DN rabbits received hemodialysis (HD) treatment every 3 days, (3) DM + CAER platform: diabetic rabbits received the CAER platform treatment every 3 days, and (4) DN + CAER platform: DN rabbits received the CAER platform treatment every 3 days.

#### 2.8.1. Oral Glucose Tolerance Test (OGTT)

Glucose tolerance was measured using the OGTT assay [28]. After fasting for 8 h, all DM/DN rabbits were intragastrically fed with 2 g/kg glucose within 2–3 min after receiving different treatments. At 0.5, 1, 1.5, and 2 h time points, a drop of blood was taken from the auricular vein and the blood glucose concentration was measured using a blood glucose meter (Yuwell, Jiangsu, China). The serum INS concentrations were measured using an Insulin ELISA kit (GSB-E05071m, Cusabio, Wuhan, China).

#### 2.8.2. Blood Biochemistry Index

During the OGTT, the H_2_O_2_ concentration was measured using an H_2_O_2_ assay kit (Bomei Biotechnology, China). Hematological parameters were determined as described previously.

#### 2.8.3. The Long-Term Effects

As the DN rabbits receiving the CAER platform treatment maintained a normal range of BGL within 72 h, the rabbits in the DM + GOx-INS@ER and DN + GOx-INS@ER groups received hemodialysis treatment every 3 days. The BGL, serum INS, CREA, and BUN were tested as previously reported.

### 2.9. Histological Observation

After the CAER platform treatment, rabbits were euthanized by CO_2_ inhalation and the main organs were harvested for histological examination. The sliced specimens were 4 mm thick, stained with hematoxylin and eosin (H&E), and observed under a DFC290 light microscope (Leica, Wetzlar, Germany).

### 2.10. Blood Biochemistry Index

An automatic biochemical analyzer (Trilogy, France) determined liver and renal function indexes such as globulin (GLB). Rabbit ELISA kits (Bomei Biotechnology, China) were used to analyze inflammatory factors (IL-1β, IL-6, and TNF-α).

### 2.11. Statistical Analysis

Two-sample t-test was used between the two groups and two-factor ANOVA was performed in SPSS 12.0 software according to three independent variables. All values were averaged and expressed as the mean ± standard deviation. A *p*-value less than 0.05 is considered statistically significant.

## 3. Results

ERs as pharmacological vehicles to deliver medicines have the advantages of prolonging the systemic action time and reducing adverse reactions. However, the limited sources for ERs restricts their clinical use. In this study, we modified the commercial hemodialysis system with a CAER platform to deliver INS, which could be clinically applied in the treatment of DM. The CAER platform was divided into three major sections: (I) INS reactor. INS was loaded into ER by the hypotonic dialysis method in this reactor. (II) GOx reactor. GOx was modified on the surface of the INS@ERs. (III) Hemodialysis. The GOx-INS@ER suspension was dialyzed to remove residual free INS and GOx and then returned to the venous blood.

### 3.1. INS@ER Assembly in the INS Reactor

**Figure 1:**
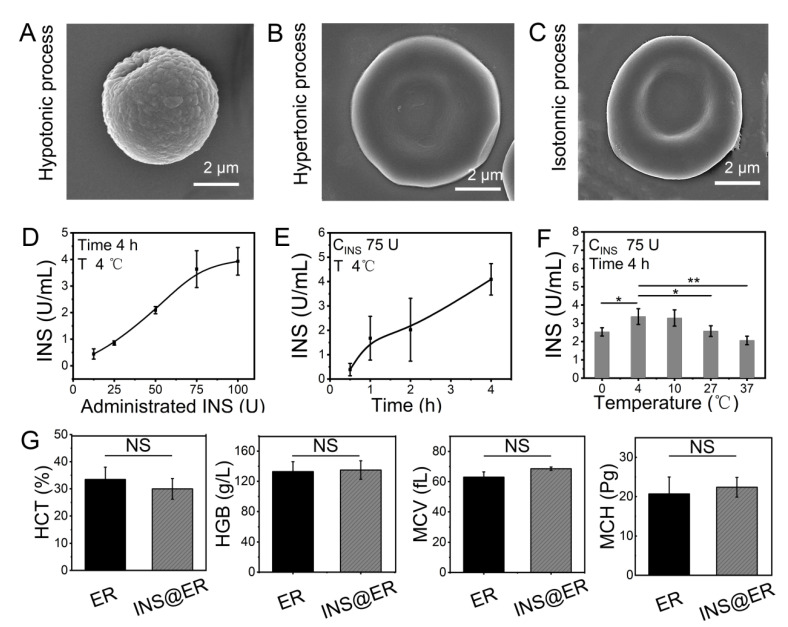
The construction of INS@ERs in the INS reactor. (**A**) SEM image of an ER in hypotonic process. (**B**) SEM image of an ER in hypertonic process. (**C**) SEM image of an ER in isotonic process. (**D**) The relationship between administrated INS content in the INS reactor and loading concentration in the INS@ERs. (**E**) The change curve of INS concentration in the INS@ERs with the hypotonic time. (**F**) The plot of the relationship between hypotonic temperature and INS concentration. * indicates *p* < 0.05, ** indicates *p* < 0.01, and NS indicates *p* > 0.05 with comparison between groups. (**G**) Hematological parameters of native ERs and INS@ERs, including HCT, HGB, MCV, and MCH. NS indicates non-significance.

The rabbit’s blood was drained via a jugular vein cannula into the INS reactor after anesthesia and heparin administration. As ERs make up more than 90 % of the total blood cells, the INS would be loaded mainly in the ERs [40,41,42]. To realize a practical ER-loading INS system, the INS loading process includes hypotonic, hypertonic, and isotonic dialysis by changing the extracellular solution. In detail, ERs and INS were dispersed in the dialysis bag of the INS reactor in a hypotonic solution and the membranes of the ERs were opened by pre-expansion (Figure 1A and Figure S3), facilitating the encapsulation of INS into ERs. Then, the hypotonic solution was siphoned off from the dialysis bag and perfused with the hypertonic solution at 37 °C to restore the double concave disc shape of the ERs (Figure 1B). After these procedures, the hypertonic solution was siphoned off and the isotonic solution was perfused for another 15 min. After these processes, INS@ERs were obtained and their morphology restored to a typical concave disk structure similar to native ERs (Figure 1C and Figure S4). The size of an INS@ER was also similar to a pristine ER (Figure S5).

For most DN patients, the hemodialysis process is about 4 h per session. So, the loading of INS in ERs should be optimized to obtain higher loading efficiency and loading content within 4 h. Thus, factors that influenced the loading of INS, such as the feeding amounts of INS, hypotonic time, and temperature, were studied. As shown in Figure 1D, the loading content of INS in ERs increased linearly with the ascendant INS concentration until it approached 75 U. Extending the incubation time resulted in higher INS loading efficacy (Figure 1E). A temperature of 4 °C was proved as the best reaction temperature in hypotonic dialysis (Figure 1F). The optimal procedure to prepare INS@ERs was witnessed with 75 U of INS at 4 °C, with 4 h of dialysis to load the maximum amount of INS. Therefore, this protocol was adopted in the INS@ER reactor to achieve high INS loading content (about 3.72 ± 0.18 U/mL). Finally, the INS encapsulation efficiency in RBC was calculated to be about 9.92 %. INS@ERs could remain stable in PBS for at least 4 days (Figure S6). Moreover, there was no statistical difference in HCT, MCV, MCH, MCHC, and other hematologic parameters between INS@ERs and normal ERs, confirming that INS@ERs could be used in vivo (Figure 1G).

### 3.2. GOx-INS@ER Assembly in the GOx Reactor

After its assembly, the INS@ER suspension was pumped into the GOx reactor. The key to this work is to modify GOx on the GOx-INS@ER’s surface to achieve glucose response characteristics. After the GOx was treated with biotin, it was added to the GOx reactor and mixed with INS@ERs for about 0.5 h. No significant changes were observed both for INS content in ERs and the ER morphology pre- and post-modification by GOx (Figure S7).

**Figure 2:**
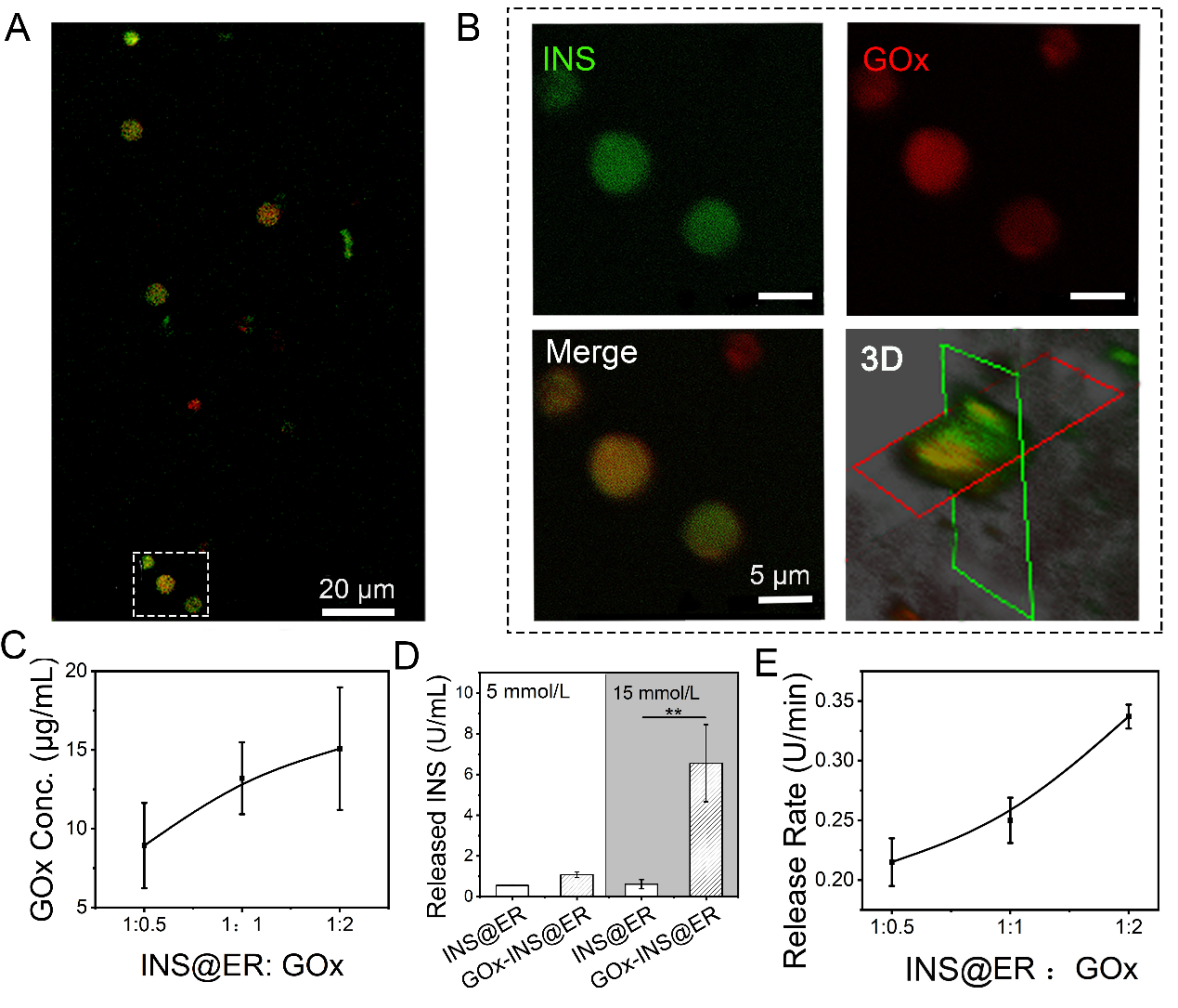
The construction of GOx-INS@ERs in the GOx reactor. (**A**) Confocal images of GOx-INS@ERs. Green: FITC-labeled INS; red: rhodamine-B-labeled biotin-GOx. (**B**) Zoom-in views of the white dotted boxed areas in Figure 2A and confocal microscopy 3D-reconstruction of GOx-INS@ERs. (**C**) The curvilinear relationship between ER and GOx ratio and the concentration (conc.) of GOx on the GOx-INS@ERs. (**D**) The INS release behavior of INS@ERs or GOx-INS@ERs in response to glucose; 5 mmol/L D-glucose: set as normal glucose level, 15 mmol/L D-glucose: set as hyperglycemic glucose level. (**E**) Relationship between different INS@ER to GOx ratios on INS release rate from GOx-INS@ERs in hyperglycemia. ** *p* < 0.01.

The presence of GOx on the INS@ER’s surface was confirmed by confocal images (Figure 2A,B). In this experiment, rhodamine-B-(red)-modified biotin-GOx was co-incubated with FITC-labeled INS@ERs. After loading FITC-INS, ERs presented green in the FITC channel, clearly indicating that INS was successfully encapsulated inside the ERs. Rhodamine-B-labeled GOx was observed at high magnification in the red channel. In the overlay image, we noticed that the ERs loaded with green FITC-INS were coated with a layer of red rhodamine-B-modified GOx. Through confocal three-dimensional analysis, the GOx was anchored to the ER’s surface in the GOx-INS@ERs. The biotinylated GOx could probably bind to the surface of ERs because of the strong affinity between the protein and biotin [28]. We observed that different volume ratios of ERs to GOx led to different GOx content in GOx-INS@ERs (Figure 2C). Meanwhile, we find that most of the ERs, drained into the CAER platform, could work as INS delivery carriers (Figure S8). There were no statistically significant differences in hematologic indexes such as HCT, MCV, MCH, and MCHC between GOx-INS@ERs and INS@ERs (Figure S9), confirming this modification procedure did not affect the properties of INS@ERs.

To examine the glucose-regulated INS release behavior of GOx-INS@ERs, INS@ERs (without GOx) were chosen as the control. The GOx-INS@ERs and INS@ERs were immersed in normoglycemic solution (5 mmol/L of glucose solution) or hyperglycemic solution (15 mmol/L of glucose solution). As shown in Figure 2D, the amount of released INS did not differ significantly between GOx-INS@ERs and INS@ERs in normoglycemic solutions. In contrast, the amount of released INS was significantly higher in the GOx-INS@ER group than that in the INS@ER group in hyperglycemic solutions (*p* < 0.01). This indicated that GOx played a key role in the regulation of the release of INS from GOx-INS@ERs. We also found that the release rate of INS from GOx-INS@ERs in hyperglycemic solution was proportional to the content of GOx (Figure 2E). In the following experiment, a 1:2 ratio of INS@ERs to GOx was selected to achieve rapid INS release. 

### 3.3. Hemodialysis of GOx-INS@ER suspension through the Hollow Fibers of the Hemodialyzer

**Figure 3:**
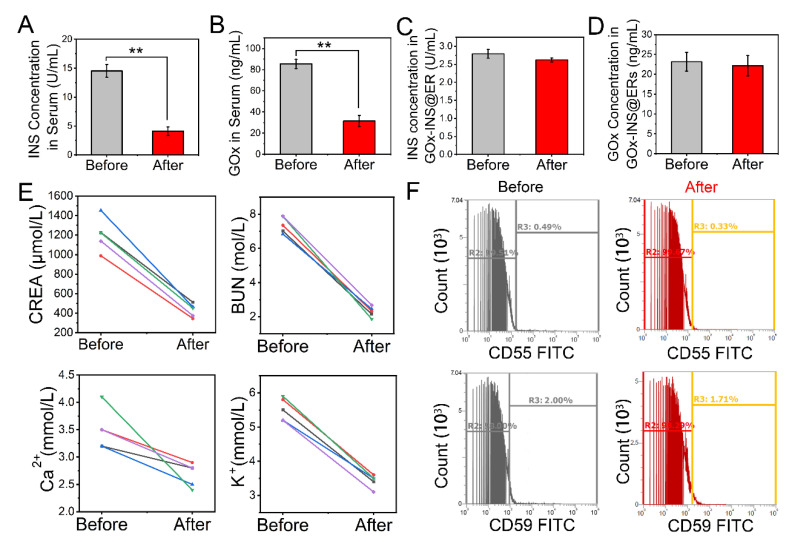
Changes in blood parameters during the hemodialysis process of the CAER platform. (**A**) Mean concentration changes in unencapsulated INS in the serum. (**B**) Mean concentration changes in unmodified GOx from GOx-INS@ERs. (**C**,**D**) Effects of hemodialysis on the INS concentration and GOx in the GOx-INS@ERs. (**E**) CREA, BUN, Ca^2+^, and K^+^ concentrations before and after hemodialysis. (**F**) Surface expression levels of CD55 and CD59 on GOx-INS@ERs and natural ERs measured by flow cytometry. ** *p* < 0.01.

Free INS and GOx would be present in the solution after the synthesis of GOx-INS@ERs. Because excessive doses of exogenous free INS can cause hypoglycemia, a purification step is necessary. To remove the free INS and GOx, the hollow fibers (Nano-A, showed in Scheme 1b IV) of the hemodialyzer acted as hemodialyzers to purify GOx-the INS@ER suspension in the CAER platform. We tested the clearance of INS and GOx before and after hemodialysis (Figure 3A,B). After passing through the hollow fibers of the hemodialyzer, free INS and GOx in the blood decreased significantly, indicating most of the free INS and GOx were removed from the blood, guaranteeing the safety of GOx-INS@ERs in vivo. Meanwhile, the amounts of INS and GOx in the GOx-INS@ERs did not change significantly before and after hemodialysis (Figure 3C,D). The hollow fibers of the hemodialyzer showed an excellent hemodialysis effect, as the CREA, BUN, K^+^, and Ca^2+^ levels decreased significantly post hemodialysis (Figure 3E). These reductions in renal indexes suggest that the CAER platform could benefit the treatment of renal disease.

The stability of ER membrane proteins after drug loading is crucial to their clinical performance, as aged ERs would be cleared prematurely [43]. We investigated the expression of antiaging proteins, CD55 (complement decay accelerating factor or DAF) and CD59 (protectin), that can prevent the formation of the membrane attack complex (Figure 3F). CD55 and CD59 expression was not decreased after drug loading, which suggested that the GOx-INS@ERs had a similar capability for long circulation in vivo as natural ERs. Furthermore, the INS in the GOx-INS@ERs retained its original biological activity (Figure S10).

### 3.4. Glucose Responsive, Closed-Loop Release of INS from GOx-INS@ERs

**Figure 4:**
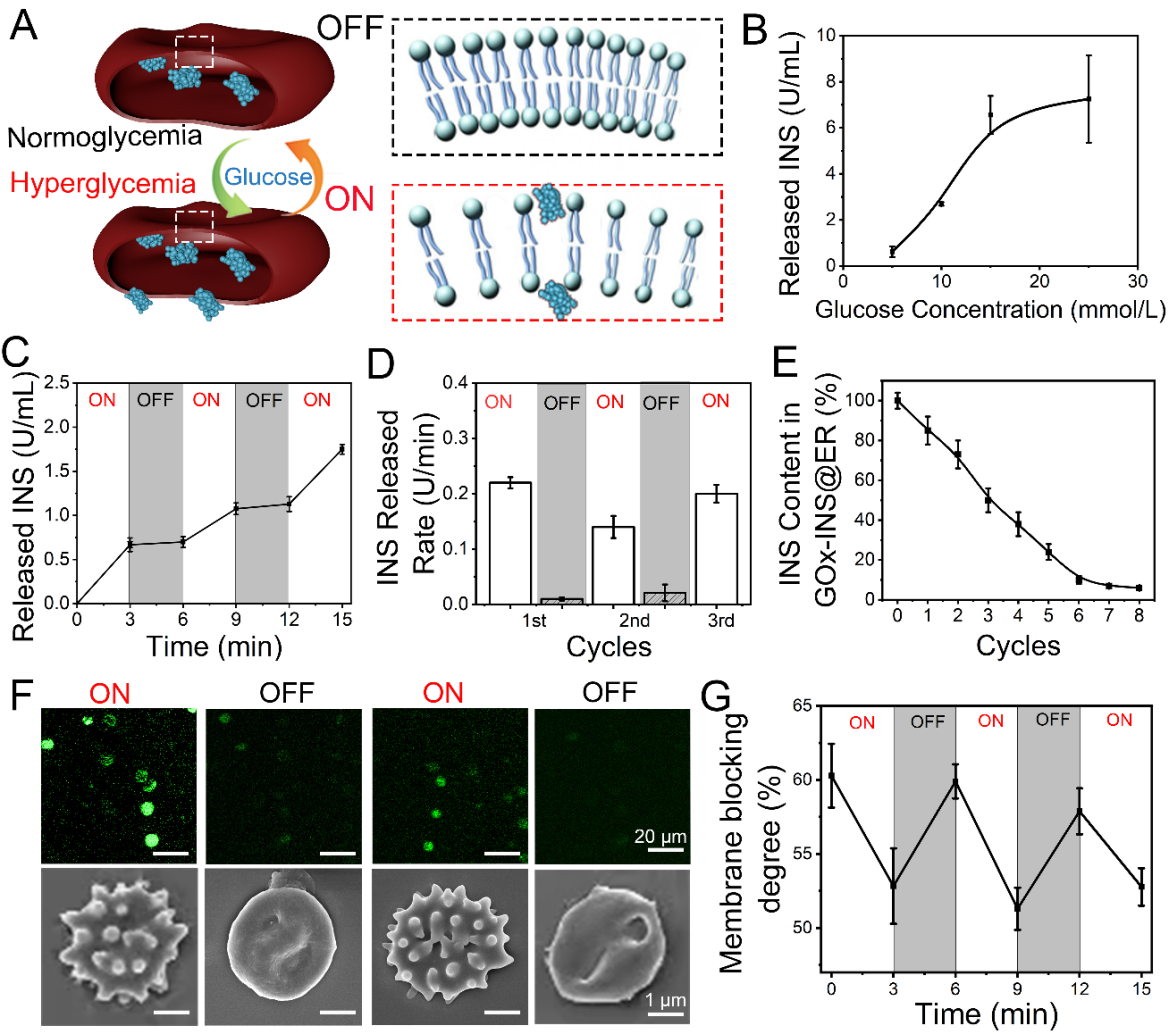
Glucose-responsive release behavior of GOx-INS@ERs (constructed via the CAER platform) in vitro. (**A**) Schematic diagram of a GOx-INS@ER’s blood glucose-responsive INS release. Under hyperglycemic conditions, INS is released from a GOx-INS@ER as the pore of the bilayer lipid membrane opens, indicating the “on” status of the GOx-INS@ER. When the blood glucose level returns to normal, the GOx-INS@ER stops INS release and maintains its spherical shape with the integrated cellular membrane (“off”). (**B**) INS release behavior of GOx-INS@ERs in different concentrations of glucose at 37 °C. (**C**) Glucose-responsive INS release behavior of GOx-INS@ERs in different glucose levels (normoglycemia, 5 mmol/L; hyperglycemia, 15 mmol/L) at 37 °C. (**D**) INS release rate of GOx-INS@ERs in “on-off” cycles at 37 °C. (**E**) The change curve of INS in GOx-INS@ERs during the INS release cycle tests. (**F**) Generation and quenching of ROS and SEM images representing the morphological changes of GOx-INS@ERs in the “on” or “off” state. (**G**) GOx-INS@ER membrane blocking degree curve during “on-off” cycles (normoglycemia, OFF; hyperglycemia, ON).

After the INS was loaded inside the ERs through the CAER platform, the glucose-activated INS release behavior of GOx-INS@ERs was proved. We had reported the use of GOx in a glucose-activated switch to control the release of INS [28]. According to our design (shown in Figure 4A), the GOx-INS@ER suspension was pumped back into the body. When the blood glucose showed normal glycemia, they were in the ‘‘off” state, working as native ERs in the blood. When the BGL rises (hyperglycemia), GOx will catalyze the conversion of glucose to gluconic acid and H_2_O_2_, which causes the rapid rupture of the ERs, creating pores and releasing INS (the “on” state).

The release of INS was positively correlated with glucose concentration (Figure 4B). As the BGL of DN rabbits should be less than 10 mmol/L [44], the GOx-INS@ERs should regulate the release of INS according to the variation in BGL. The glucose responsive INS release behavior of the GOx-INS@ERs was tested by incubating them in normoglycemic (5 mmol/L) or hyperglycemic solutions (15 mmol/L). As shown in Figure 4C, the INS was rapidly released when the GOx-INS@ERs were incubated in a hyperglycemic solution (15 mmol/L). In contrast, INS release was stopped when the glucose level was reduced to the normal blood glucose level (5 mmol/L). The INS release rate from GOx-INS@ERs in hyperglycemic solution (“on”) was much faster than that in normoglycemic solution (“off”) (Figure 4D). Additionally, total amount of INS in GOx-INS@ERs decreased upon the repeating of the on/off cycles (Figure 4E).

As ROS played a vital role in the glucose-responsive release of INS from GOx-INS@ERs, a ROS-sensitive fluorescent probe (DCFH-DA) was used to study the release mechanism (Figure 4F). The fluorescence intensity of the ROS was enhanced in the hyperglycemic solution when the GOx-INS@ERs were in an “on” state. The SEM images also revealed that the GOx-INS@ERs had irregular surfaces in the hyperglycemic solution. When the GOx-INS@ERs were in the “off” state, the fluorescence intensity of the ROS decreased and the GOx-INS@ERs returned to normal morphologies. Furthermore, the GOx-INS@ER membrane blocking degree as an index of release was evaluated (Figure 4G). The membrane blocking degree was consistent with the changes in glucose levels, reflecting the glucose-activated INS release behavior of GOx-INS@ERs.

Based on the data in Figure 4, the GOx-catalyzed oxidation of glucose produces ROS that worked as the optical switch to be used in the self-regulated INS-releasing behavior. Under hyperglycemia, excessive glucose increased ROS production, which promoted pore formation on ER membranes, allowing the release of INS. The released INS reduced the BGL to the normal range, at which time the production of ROS was greatly suppressed. Together, the excess ROS was scavenged by superoxide dismutase (SOD) and catalase (CAT), which led to the closure of the pores on ER membranes, stopping the INS release.

### 3.5. Controlling the BGL in DN Rabbits

**Figure 5:**
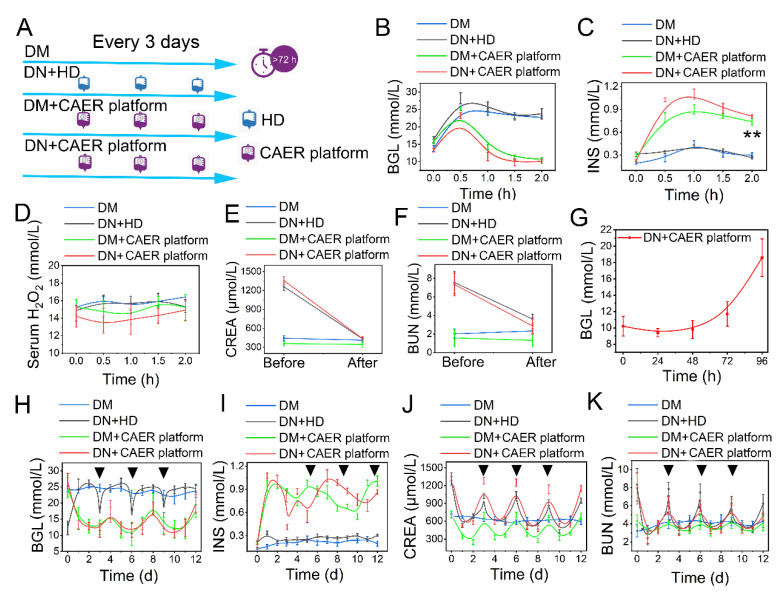
Evaluation of glycemic control and dialysis effect of the CAER platform. (**A**) Schematic of experimental design. The rabbits were randomly allocated into four groups and received different treatments: (1) DM: diabetic rabbits, set as control. (2) DN + HD: DN rabbits received hemodialysis (HD) treatment every 3 days. (3) DM + CAER platform: diabetic rabbits received the CAER platform treatment every 3 days. (4) DN + CAER platform: DN rabbits received the CAER platform treatment every 3 days. (**B**–**F**) The oral glucose tolerance test results in vivo. (**B**) The BGL curve. (**C**) The change curve of serum INS concentration. (**D**) The curve of serum H_2_O_2_ concentration. (**E**) Change in serum CREA concentration. (**F**) Concentration changes of BUN in serum. (**G**) The curve of blood glucose level after one treatment. (**H**–**J**) The results of long treatment (once every three days). The black tringles indicated the treatment time. (**H**) The curve of blood glucose level after multiple treatments. (**I**) The change curve of blood INS concentration after multiple treatments. (**J**) CREA concentration changes. (**K**) Concentration changes of BUN in serum after multiple treatments. ** *p* < 0.01.

DN rabbits were used to investigate the possibility of controlling the DN with the CAER platform. The treatment schedule is illustrated in Figure 5A. We performed the OGTT experiment reference standard to test the therapeutic efficacy of the CAER platform [45]. After receiving various types of treatment, all rabbits were fed glucose at a dose of 2 g/kg. We monitored the changes in BGL for 2 h. The BGL in DM and DN + HD groups went up immediately and did not descend with over time because of the deficiency in endogenous INS (Figure 5B,C). Contrarily, the BGL in the DM + CAER platform and DN + CAER platform groups slowly reduced to normal levels just as normal rabbits did (Figure 5B and Figure S11). The serum INS levels in DM + CAER platform and DN + CAER platform groups were as high as in normal rabbits (Figure 5C and Figure S12). This illustrates that the GOx-INS@ERs in the CAER platform could respond to the hyperglycemic environment and release INS. As GOx on the GOx-INS@ERs catalyzed the conversion of glucose to H_2_O_2_, we monitored H_2_O_2_ levels in the serum after CAER platform treatment. As shown in Figure 5D and Figure S13, H_2_O_2_ levels were stable and similar to those in normal rabbits. Based on these results, we speculated that the H_2_O_2_ on the surface of the GOx-INS@ERs could work as the optical switch to be used in the self-regulated INS-releasing behavior. Once H_2_O_2_ escaped from the GOx-INS@ERs and entered the blood, it would be decomposed to water and oxygen by enzymes (such as SOD and CAT), thus avoiding damage to the healthy tissues [28,46,47]. Meanwhile, CREA and BUN were measured before and after the treatment with the CAER platform (Figure 5E,F). The levels of CREA and BUN in the DN + CAER platform group decreased to normal values, indicating the good hemodialysis function of the CAER platform.

The DN rabbits receiving the CAER platform treatment could maintain their BGLs in the normal range for 72 h (Figure 5G). In the long-term treatment study, the frequency of the CAER platform treatment was every 3 days in the DM + CAER platform and DN + CAER platform groups. In the following 12 days, as shown in Figure 5H and Figure S14, the BGLs in DM + CAER platform and DN + CAER platform groups were same as in the normal rabbits. Hemodialysis could control the BGL transiently in the DN + HD group. The in vivo concentration of INS in the CAER platform treatment group was higher than that in DM and DN + HD groups (Figure 5I and Figure S15). Levels of CREA, BUN, Ca^2+^, and K^+^ were also well controlled after the CAER platform treatment (Figure 5J,K and Figure S16). The body weight and food intake results of different groups are same as for the healthy rabbits, as shown in Figure S17. The above results indicate that the CAER platform has both good glycemic control and hemodialysis functions.

### 3.6. The In Vivo Safety of the CAER Platform Treatment

**Figure 6:**
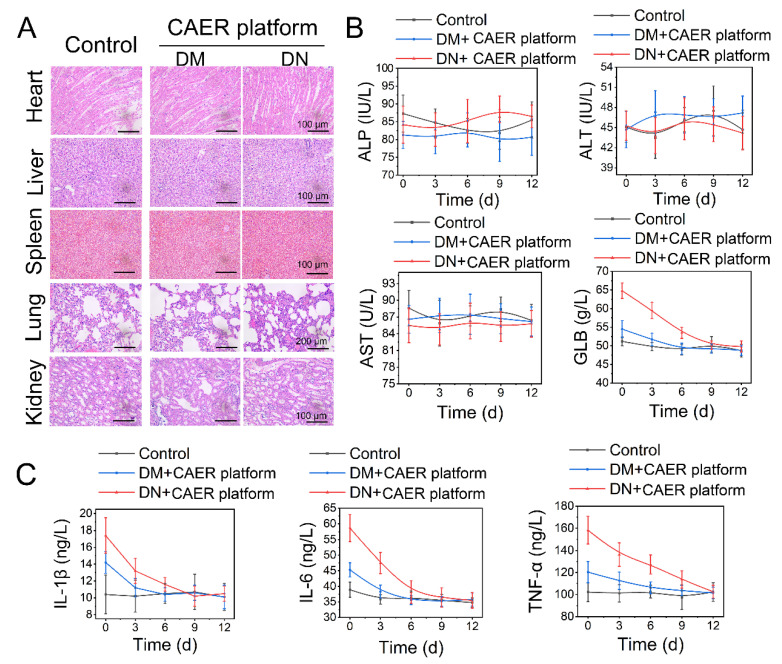
Biosafety of the CAER platform. (**A**) H&E staining of sections of the main organs, including heart, liver, kidney, lung, and spleen, after the CAER platform treatment. (**B**) Parameters of serum biochemistry profiles. (**C**) The degree of inflammation factors, including IL-1β, IL-6, and TNF-α.

The treated diabetic rabbits were euthanized after 12 days to assess the potential health risks after treatment with the CAER platform. Heart, liver, kidney, lung, and spleen tissues were stained with H&E (Figure 6A). No detectable lesions, such as inflammation, hydropic degeneration, or pulmonary fibrosis, were found in tissue sections, suggesting that the CAER platform did not damage these organs. Moreover, the liver and kidney function indexes revealed that the CAER platform did not cause hepatic and kidney damage (Figure 6B).

After the DM and DN rabbits were treated with the CAER platform for 12 days, TNF-α, IL-1β, and IL-6 levels declined steadily (Figure 6C). This indicated that inflammation and other adverse reactions caused by hyperglycemia were suppressed after the CAER platform treatment. These results show that this treatment exhibits good biocompatibility in vivo and can aid the therapy of DM and DN in rabbits.

## 4. Conclusions

In this study, we report that a CAER platform using autologous ERs as INS-carriers and GOx expressed on the surface of the cells to function as a glucose-activated switch could accomplish glycemic control and hemodialysis for diabetes mellitus therapy. This CAER platform was an autologous ER-mediated delivery platform that could overcome the limited ER sources and decrease the risk of transmitting infections, accomplishing the construction of GOx-INS@ERs based on a commercial hemodialysis instrument. The GOx-INS@ERs could release INS upon a change in BGL, overcome hyperglycemia within 2 h, and maintain the BGL at a normal level for up to 3 days. The results from in vivo experiments in a DN rabbit model show that this CAER platform could control the BGL well and complete hemodialysis. Therefore, this CAER platform has the promise to be used as a potential treatment for DN in the clinic.

## Data Availability

Not applicable.

## Supplementary Materials

The following supporting information can be downloaded at: https://www.mdpi.com/article/10.3390/nano12203556/s1, Figure S1: Schematic representation of the in vitro, close-looped autologous ER-mediated delivery (CAER) platform; Figure S2: The diagram of the insulin (INS) reactor design; Figure S3: Representative flow cytometry results; Figure S4: The SEM image of native ER; Figure S5: The size changes statistical graph during the preparation of INS@ER; Figure S6: The stability of INS@ER, including the changes of INS concentration and hematological parameters of INS@ER; Figure S7: The INS concentration changes and morphology image during GOx-INS@ER assembly in the GOx reactor; Figure S8: Hematological parameters change during the GOx-INS@ER assembly; Figure S9: The curve of blood glucose level (BGL) of normal rabbits in vivo in the oral glucose tolerance test; Figure S10: The change curve of serum INS concentration in normal rabbit group; Figure S11: The curve of serum H_2_O_2_ concentration in normal rabbit group; Figure S12: The curves of blood glucose unit (GLU) of normal rabbits, which were fed with a normal diet; Figure S13: The curves of serum INS concentration of normal rabbits, which were fed with a normal diet; Figure S14: The change curves of Ca ions and potassium ions of the different treatment groups. Figure S15: The body weight and food intake results in different group; Figure S16: The change curves of Ca ions and potassium ions of the different treatment groups. DM: diabetic rabbits, set as control. DN+HD: DN rabbits received hemodialysis (HD) treatment every 3 days. DM+ CAER platform: diabetic rabbits received the CAER platform treatment every 3 days, and DN+ CAER platform: DN rabbits received the CAER platform treatment every 3 days; Figure S17: The body weight and food intake results in different group. Healthy rabbits: set as normal control. DM: diabetic rabbits, set as positive control. DN + HD: DN rabbits received hemodialysis (HD) treatment every 3 days. DM + CAER platform: diabetic rabbits received the CAER platform treatment every 3 days, and DN + CAER platform: DN rabbits received the CAER platform treatment every 3 days; Table S1: Recipes of the buffer solutions used in the INS reactor.
