# Supplementary material for: A Closed-Loop Autologous Erythrocyte-Mediated Delivery Platform for Diabetic Nephropathy Therapy
Source: Nanomaterials (Basel). 2022 Oct 11;12(20):3556. doi: 10.3390/nano12203556 (PMC9612375; doi:10.3390/nano12203556)
Supplement: Supplementary file 1 [file nanomaterials-12-03556-s001.zip › nanomaterials-1903071-supplementary.pdf]

Supplementary Materials

A Closed-Loop Autologous Erythrocyte-Mediated Delivery Platform for Diabetic Nephropathy Therapy

Lingzi Feng [1,†], Xinzhong Huang [2,†], Jia Li [1], Chao Chen [1], Yidan Ma [2], Haiying Gu [1,*], Yong Hu [3,*] and Donglin Xia [1,*]

[1] School of Public Health, Nantong University, Nantong 226019, China
[2] Department of Nephrology, Affiliated Hospital of Nantong University, Nantong 226001, China
[3] College of Engineering and Applied Sciences, MOE Key Laboratory of High Performance Polymer Materials & Technology, Nanjing University, Nanjing 210033, China
* Correspondence: hygu@ntu.edu.cn (H.G.); hvyong@nju.edu.cn (Y.H.); xiadonglin@ntu.edu.cn (D.X.)
† These authors contributed equally to this work.

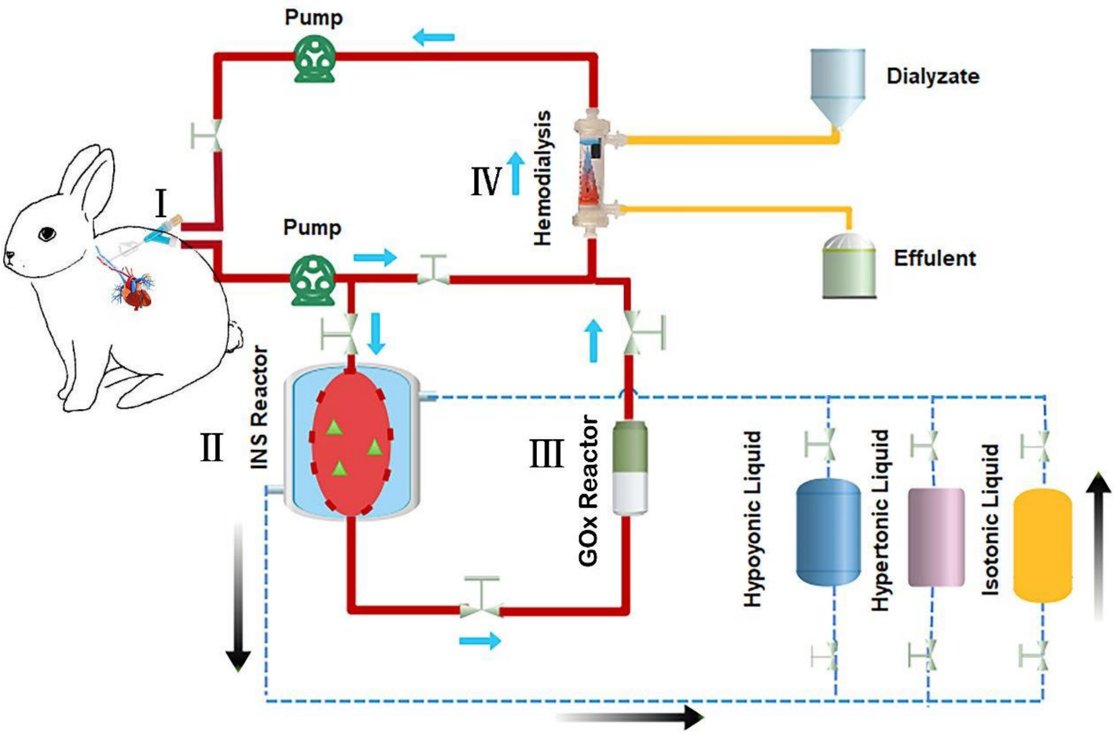

Figure S1. Schematic representation of the *in vitro*, close-looped autologous ER-mediated delivery (CAER) platform. The schematic representation shows the CAER platform, accomplished the construction of a glucose-responsive, self-regulated INS release system (GOx-INS@ER). (I), Blood drive. Blood was drained via a jugular vein cannula and eventually back into the animal vein. (II), INS@ER assembly. INS was loaded into ER in the INS reactor by the hypotonic dialysis method. (III), GOx-INS@ER assembly. GOx was modified on the surface of the INS@ER in the GOx reactor. (IV), Hemodialysis. GOx-INS@ER was dialyzed to remove residual drugs before returned to the venous blood.

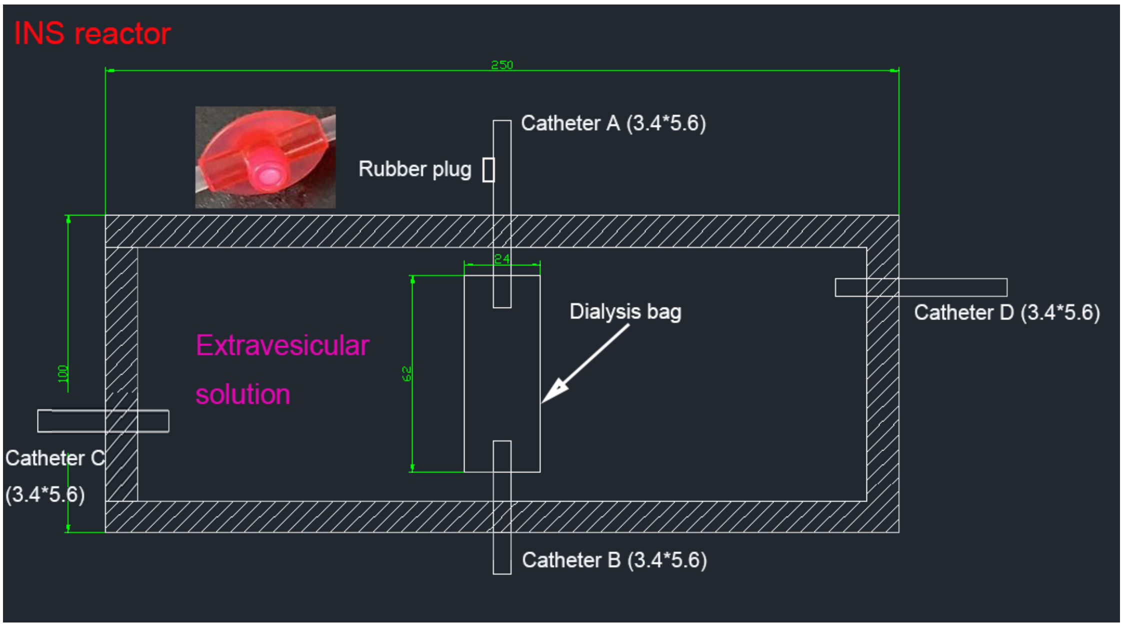

Figure S2. The diagram of the insulin (INS) reactor design. The blood could be drained into the INS reactor through Catheter A, and drained out through Catheter B, while the hypotonic buffer, hypertonic solution, or isotonic solution could be perfused in through Catheter C and out though Catheter D. Inset: the real product picture of rubber plug for the addition of INS.

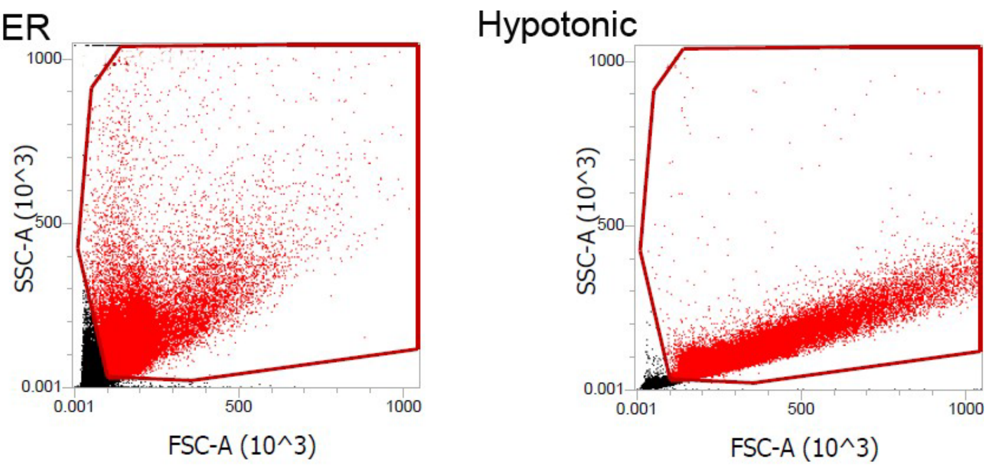

Figure S3. Representative flow cytometry result. FSC is indicative of cell size. In hypotonic solution, the size of ER became larger by pre-expansion.

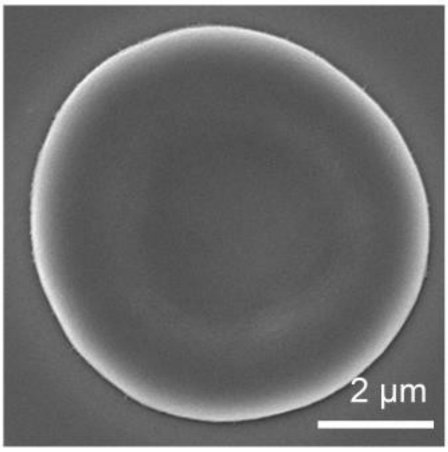

Figure S4. The SEM image of native ER.

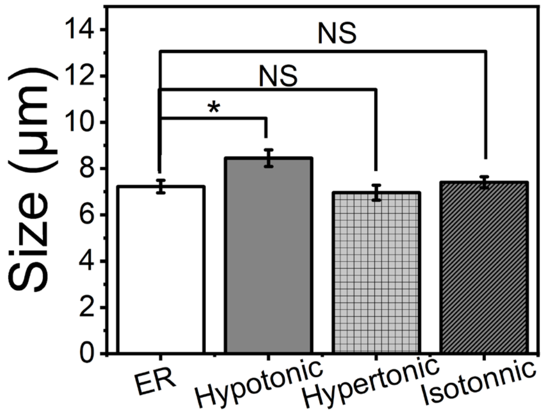

Figure S5. The size changes statistical graph during the preparation of INS@ER.
* Indicate P < 0.05, NS indicates non-significance.

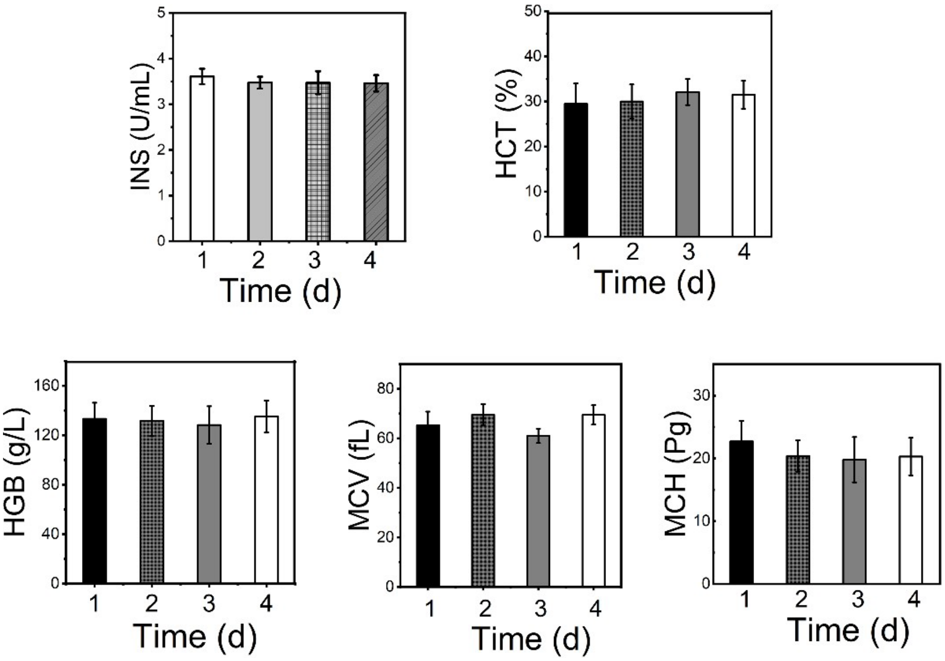

Figure S6. The stability of INS@ER, including the changes of INS concentration and hematological parameters of INS@ER, such as hematocrit (HCT), hemoglobin (HGB), mean corpuscular volume (MCV), and mean corpuscular hemoglobin (MCH).

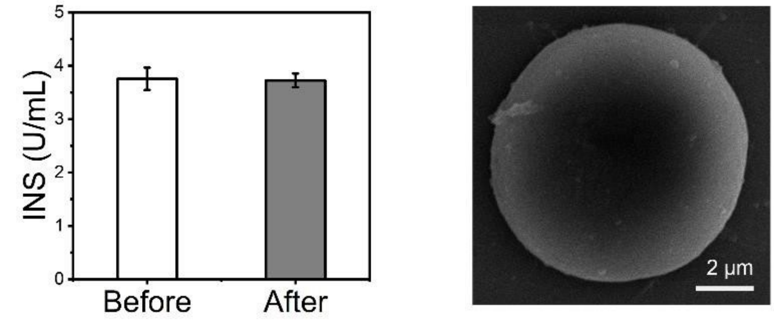

Figure S7. The INS concentration changes and morphology image during GOx-INS@ER assembly in the GOx reactor. No significant changes were observed both for INS content in ER and the ER morphology pre and post-modification by GOx.

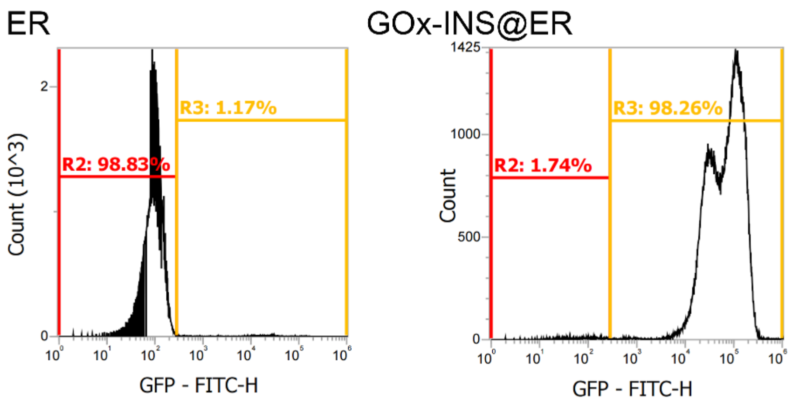

Figure S8. Representative flow cytometry results of ER loaded with FITC-INS. Flow cytometric analysis demonstrated that >98% of the ER expressed FITC in the CAER platform. This revealed that most of ER, drained into the CAER platform, could work as INS delivery carriers.

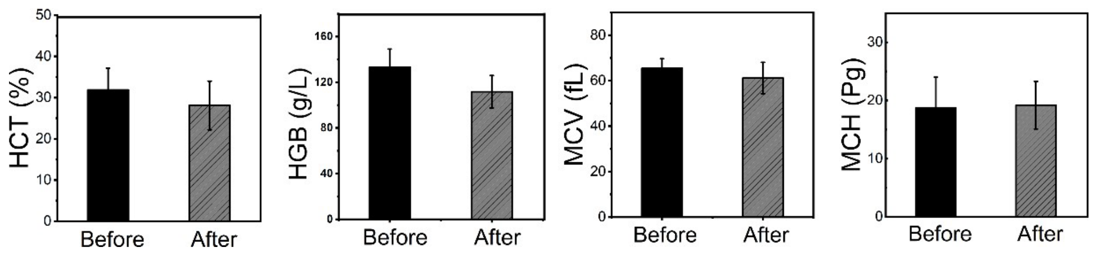

Figure S9. Hematological parameters change during the GOx-INS@ER assembly, including Hematocrit value (HCT), hemoglobin concentration (HGB), mean corpuscular volume (MCV), and mean corpuscular hemoglobin (MCH). There were no statistically significant differences in hematologic indexes like HCT, MCV, MCH, and MCHC between GOx-INS@ER and INS@ER.

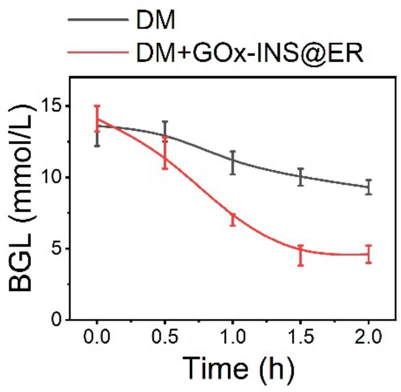

Figure S10. The curve of blood glucose level (BGL) of DM rabbits treated with 0.5 mL GOx-INS@ER, was drawn from the CAER platform. The BGL decreased to the normal range within 2 h, which indicated that the INS in the GOx-INS@ER retained its original biological activity.

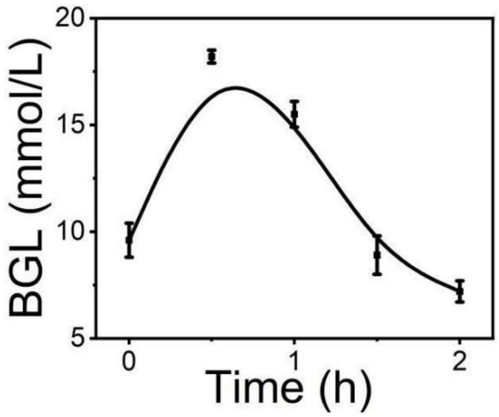

Figure S11. The curve of blood glucose level (BGL) of normal rabbits *in vivo* in the oral glucose tolerance test. The BGL rapidly increased after the oral administration of glucose and return to normal within 2 h.

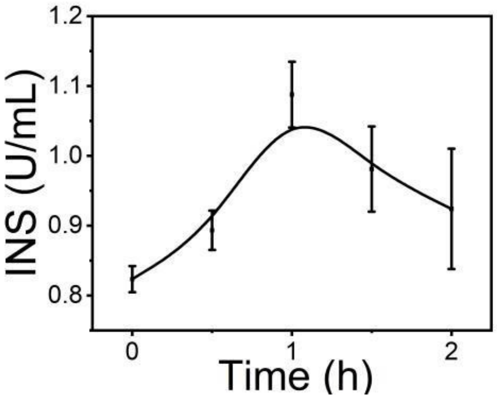

Figure S12. The change curve of serum INS concentration in normal rabbit group. INS secretion level is increased as blood glucose elevated.

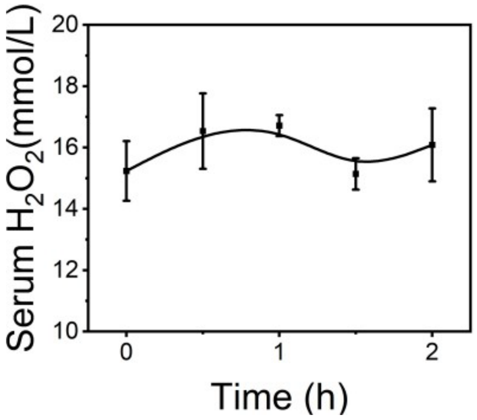

Figure S13. The curve of serum H_2O_2 concentration in normal rabbit group.

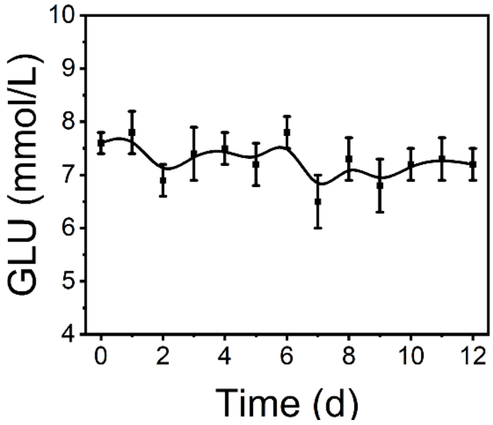

Figure S14. The curves of blood glucose unit (GLU) of normal rabbits, which were fed with a normal diet.

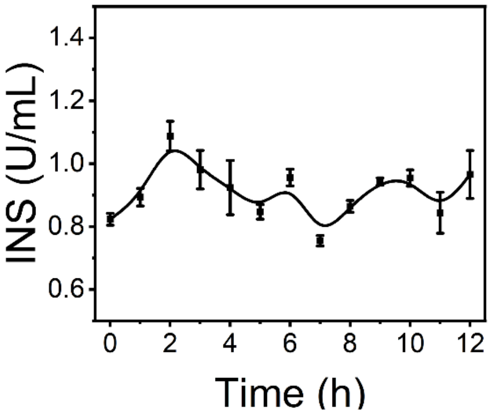

Figure S15. The curves of serum INS concentration of normal rabbits, which were fed with a normal diet.

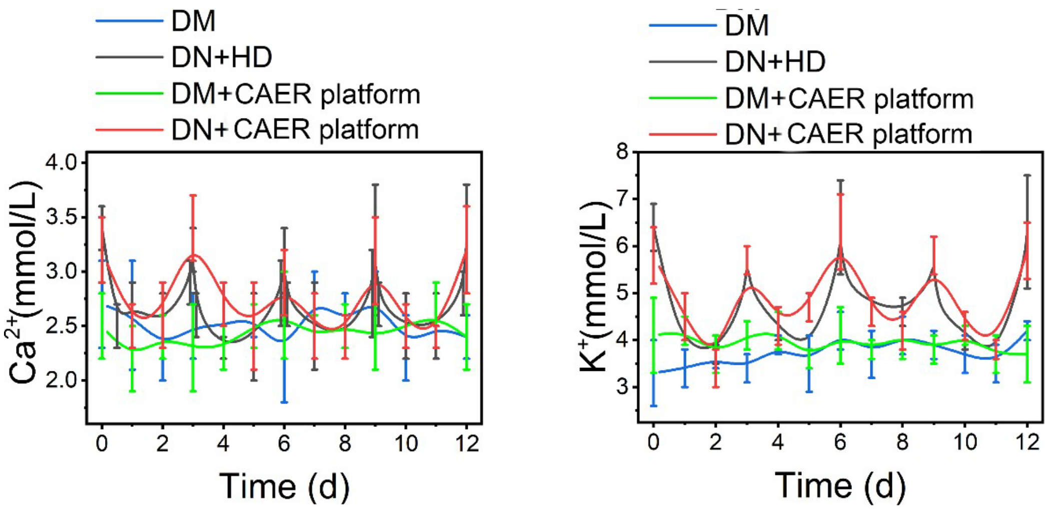

Figure S16. The change curves of Ca ions and potassium ions of the different treatment groups. DM: diabetic rabbits, set as control. DN+HD: DN rabbits received hemodialysis (HD) treatment every 3 days. DM+ CAER platform: diabetic rabbits received the CAER platform treatment every 3 days, and DN+ CAER platform: DN rabbits received the CAER platform treatment every 3 days.

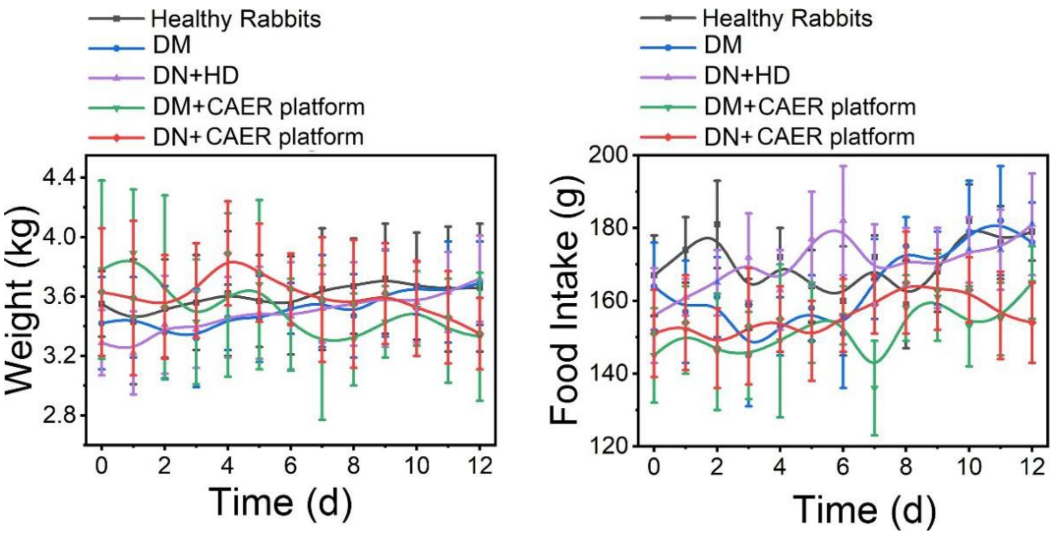

Figure S17. The body weight and food intake results in different group. Healthy rabbits: set as normal control. DM: diabetic rabbits, set as positive control. DN + HD: DN rabbits received hemodialysis (HD) treatment every 3 days. DM + CAER platform: diabetic rabbits received the CAER platform treatment every 3 days, and DN + CAER platform: DN rabbits received the CAER platform treatment every 3 days.

Table S1. Recipes of the buffer solutions used in the INS reactor.

Salt	hypotonic buffer (mM)	hypertonic solution(mM)	isotonic solution (mM)
$NaH_2PO_4\,2H_2O$	15	12.5	
$NaHCO_3$	15		
ATP	2		
glutathione	3		
glucose	20	12.5	5
NaCl	5	250	150
sodium pyruvate		12.5	
inosine		12.5	
adenine		0.63	
KCl			5
$MgCl_2$			1.2
$CaCl_2$			2
HEPES			10
**pH(adjust)**	8.0	8.0	7.2
**Osmotic pressure**	72 mOsm/kg	550 mOsm/kg	300 mOsm/kg
